# Text-to-image models reveal specific color-emotion associations

**DOI:** 10.3389/fpsyg.2025.1593928

**Published:** 2025-06-13

**Authors:** Jorge Alvarado

**Affiliations:** Department of Industrial Engineering, Pontificia Universidad Javeriana, Bogotá, Colombia

**Keywords:** color, emotion, diffusion models, text-to-image, sensory associations

## Abstract

Text-to-image models learn associations between human-provided image tags and image features over billions of examples. As a result, such models provide a powerful mean to study the psychological relationships between colors and emotions. We generated images for different emotions descriptions varying in valence, arousal and dominance across several subjects and then extracted color features (chroma and L*a*b* values) from the resultant images to find color-emotion associations. Results show a joint effect of red and chroma to generate effects of joy, rage and negative powerless. In addition, lightness is key in generating effects of serenity, threat and a relief/stress divergence. Dominance emerged as an important dimension to understand interactions and nuances in color-emotion associations. The study highlights that specific combination of color elements convey emotions, rather than and beyond simple associations such as red-anger or lightness-valence.

## 1 Introduction

Understanding how colors evoke emotions is important to cognitive psychology and has significant implications for both research and practice. Over the past century, the investigation of color–emotion associations has provided critical insights into the interplay between sensory perception and emotional experience. While many findings support the view that the emotional impact of color is context dependent—with the same hue evoking divergent feelings depending on situational cues [e.g., red on a face eliciting anger (Young et al., 2013), red signaling failure in competitive settings (Elliot et al., 2007), or red embodying love in fashion contexts like clothing (Pazda et al., 2023; Elliot and Niesta, 2008)] there is also evidence suggesting the existence of more general color-emotion associations. For instance, the ecological valence theory posits that color–emotion links become pervasive when grounded in ubiquitous human experiences (Palmer and Schloss, 2010), and research in cross modal correspondences has highlighted that shared features such as intensity may provide a structural basis for color-emotion associations (Spence, 2011).

The precise nature of general color-emotion associations remains elusive. Over the past century, numerous experiments have explored the relationships between color and emotion, using various color dimensions and emotional frameworks. Most research usually relies on words, color patches, and sometimes carefully selected images to elicit emotional responses. New tools, such as text-to-image models for image generation, offer promising avenues for further exploration. Text-to-image models can aggregate tags-including emotional tags-assigned by diverse individuals across the globe to specific images, providing new opportunities to better understand the relationship between color variables and emotional responses.

This introduction is organized as follows. First, a review of the color and emotion frameworks used in this research is presented. Second, previous studies on color-emotion associations are discussed, categorized by the elements of the emotional frameworks. Finally, the potential of text-to-image models for investigating color-emotion associations is outlined, followed by the key research questions.

### 1.1 Color framework

According to the International Commission on Illumination (CIE), color is composed of three main dimensions: Hue, brightness/lightness and colorfulness/saturation/chroma (CIE, 2024a). Lightness and chroma are partially independent of human perception, since lightness is directly connected with the physical property of reflectance (Arend, 1993) and chroma with the spectral properties of light, such as wavelength and purity (CIE, 2024c). The distinction between brightness and lightness is particularly important when illumination varies or is non-uniform. Under conditions where no visible illumination component is present, lightness and brightness are closely related (Kingdom, 2011). Similarly, the distinction between saturation and chroma is important at high levels of brightness (CIE, 2024b). In other cases, saturation and chroma are highly correlated. The color framework of the present research is CIELAB, where L* stands for lightness, a* and b* stands for hue axes, being a* an axe of red-blue hues and b* an axe of yellow-green hues. Chroma is calculated from the a* and b* values. This way, we obtain the three main dimensions of color. The CIELAB framework was selected for being widely used and closer to a perceptual perspective of color than RGB, HSL or HSV. Also, it matches the main dimensions generated by correspondence analysis when color-emotion associations are elicited (Hanada, 2018).

### 1.2 Emotion frameworks

There are two widely used emotional frameworks: dimensional and discrete models. The dimensional framework conceives emotions as being composed of three dimensions: valence, arousal and dominance (Russell, 1980). Dimensions were constructed based on the semantic differential technique (Osgood, 1964; Russell and Mehrabian, 1977). Mehrabian and Russell (1974) defined valence as a continuum of responses ranging from positive to negative, represented with polar adjectives such as *joyful* and *unhappy*. Arousal was conceptualized as a spectrum of mental states, from low to high excitement, captured by terms like *stimulated-relaxed*. Finally, dominance was associated with notions of control and personal agency, reflected in adjectives such as *brave-discouraged.* Combinations of these three dimensions allow for a wide range of emotional experiences.

The discrete framework, on the other hand, posits that each emotion is basic, non-separable and driven by an evolutionary response to specific needs (e.g., fear for harm protection or disgust for avoiding toxins). One of the most influential models in this framework is Ekman (1971) which identifies six universally recognized basic emotions: anger, disgust, happiness, sadness, fear and surprise.

Both the dimensional and discrete frameworks are complementary and offer valuable insights into emotional functioning (Harmon-Jones et al., 2017). To summarize findings on color-emotion associations, previous research is presented according to these two emotional frameworks, the dimensional and the discrete one.

### 1.3 Color—arousal associations

Elliot (2015, 2019) summarized the research on the psychological and physiological arousal generated by color. The findings indicate that red hues are associated with higher arousal, increased chroma/saturation correlates with heightened arousal, and blue/green hues are linked to lower arousal at the psychological level. However, relationships between arousal and factors such as lightness, yellow, or orange hues remain unsupported. Studies continue to confirm the associations between red, chroma, and arousal (Pazda et al., 2024; Buechner and Maier, 2016). However, physiological studies have been less conclusive regarding the relationship between arousal and hue, as Elliot (2015, 2019) also noted.

Recent research has pointed to possible interactions of the color dimensions regarding arousal. For example, Weijs et al. (2023) found no direct effect of saturation on arousal. Instead, their findings revealed an interaction: higher arousal occurred in red compared to blue, but only under conditions of high saturation and low lightness. Additionally, they reported an inverse relationship between lightness and arousal as measured by heart rate. Similarly, Oh and Park (2022) found that stress measures related to arousal increased under conditions of high chroma and low lightness, further supporting the idea of an interaction between color elements and physiological arousal. Furthermore, an important study on psychological arousal also supports the interaction hypothesis. Wilms and Oberfeld (2018) found that only under conditions of high saturation did increased brightness affect arousal.

In conclusion, while red and saturation generally lead to higher arousal, there are potential interactions between the three color dimensions—hue, saturation, and lightness—that influence arousal, though these interactions are not yet fully understood.

### 1.4 Color—valence associations

Research on the relationship between valence and brightness consistently supports the idea that higher brightness is associated with higher valence. Zhang et al. (2016) found that participants’ elicited emotions influenced their perception of brightness in rooms and on computer screens. Specifically, emotions associated with happiness led to perceptions of increased brightness, while sadness had the opposite effect. Similarly, Wisessing et al. (2020) observed this pattern when altering the brightness of cartoon characters’ faces, although the effect was not present for emotions like anger and fear.

Additional studies provide further evidence of this valence-brightness relationship. For instance, judgments about words presented in white fonts were associated with more positive valence, while black fonts facilitated more negative valence judgments (Okubo and Ishikawa, 2011). Lakens et al. (2013) also found that brighter images were evaluated more positively, whereas darker images were perceived negatively. Pale (i.e., brighter) colors in the environment made a group of students to assess specific learning tasks as more pleasant, calmed and relaxed (AL-Ayash et al., 2016).

Moreover, Specker et al. (2018) argued that this relationship between brightness and valence is both automatic and universal. Their cross-cultural study, which controlled for hue and saturation, involved implicit and explicit associations between brightness levels and positive or negative adjectives. The pattern—positive emotions with higher brightness and negative emotions with lower brightness—emerged even more quickly in implicit associations across two countries, Japan and Austria.

Additionally, evidence suggests that chroma increases valence. The classic study by Valdez and Mehrabian (1994), showed a positive relationship of both, brightness and saturation, on valence responses to color using semantic differentials, with brightness having a stronger effect. Kaya and Epps (2004) found that achromatic colors where rated more negatively than chromatic ones. Other research has proposed a mediating effect of saturation in relationships between music and color (Palmer et al., 2013) and also in music –odor associations (Schifferstein and Tanudjaja, 2004).

However, interactions between color dimensions and emotional dimensions have also emerged. Wilms and Oberfeld (2018) working with color patches, found that under conditions of low saturation, brightness increased valence, but the effect disappeared in high saturation. Additionally, in low saturation conditions, red increased valence compared to blue, but the effect reversed in high saturation.

In another study, Pazda et al. (2024) conducted a series of experiments using selected images with varying valence and arousal values to test the associations between colorfulness and emotional dimensions. The first three experiments demonstrated that chroma increased with valence. In a fourth experiment, participants adjusted images to be either positive or negative, with four possible valence-arousal combinations. For images adjusted to be positive, those with higher valence and arousal saw the largest increase in chroma, while those with low valence and high arousal received the smallest chroma increment. For images adjusted to be negative, the largest reduction in chroma was seen for high valence and low arousal, with the smallest reduction observed for low valence and low arousal.

Regarding hue, the potential confusion between lightness and hue complicates drawing clear conclusions from the literature. Contradictory findings have been reported, with some studies showing blue as higher in valence (Valdez and Mehrabian, 1994) while others indicate yellow as higher in valence (Dael et al., 2016; Palmer et al., 2013), or an increase in valence with yellow without a corresponding decrease in the blue range (Takei and Imaizumi, 2022). Furthermore, in a recent study, Schloss et al. (2020) found that blue does not consistently associate with negative valence (such as sadness) when controlling for chroma and lightness. In fact, blue can even be linked to positive valence under certain combinations of chroma and lightness.

In summary, brightness tends to increase valence, but likely in interactions with arousal, chroma, and hue. In addition, confounding factors between hue and lightness complicate the analysis.

### 1.5 Color—dominance associations

Research on dominance/potency is relatively sparse and often context-specific [e.g., in relation to faces, where is strongly stablished (Stephen et al., 2012; Setchell and Jean Wickings, 2005) or clothing (Wiedemann et al., 2015)], which limits the ability to detect general patterns of associations between dominance and color elements. Valdez and Mehrabian (1994) found that saturation positively correlated with dominance, while brightness negatively correlated, with weak associations found for hue. Chung and Saini demonstrated that, after controlling for hue and saturation, decreasing lightness led participants to rate a product as higher in a hierarchy, a relationship they found to be mediated by dominance (Chung and Saini, 2022).

Mentzel et al. (2017) tested implicit associations between words shown in different colors and their connection to dominance. They found that red words were easily classified as related to dominance, while blue words were not strongly associated with dominance. Soriano and Valenzuela (2009), also working with words, found no main effect of hue on dominance but did observe specific interactions, weakly linking red with high dominance and yellow with low dominance.

Though limited, existing evidence suggests an inverse correlation between brightness and dominance, with a preference for associating reddish hues with dominance. However, interactions between color elements may further complicate this relationship.

### 1.6 Color—discrete emotion associations

Of Ekman’s six discrete emotions, happiness and sadness are often tested as a pair and have been used to examine valence-color relationships. These associations were discussed in the previous section on valence. Among the remaining four emotions, the most commonly tested relationship is between red and anger. Several studies have demonstrated the connection of red and anger. For instance, identifying anger as opposed to happiness was facilitated by a red background in word tasks (Fetterman et al., 2012), and red also helped distinguish anger from fear (Young et al., 2013). Additionally, individuals primed with anger were more likely to perceive red colors (Fetterman et al., 2011). Furthermore, two cross-cultural studies have confirmed this association between red and anger, although they also found that anger was linked to black (Hupka et al., 1997; Jonauskaite et al., 2020a).

Many-to-many hue-emotion relationships are not exclusive to anger. For example, black is not only associated with anger but is also linked to fear or sadness (Hupka et al., 1997), while gray is often tied to sadness. Red, similarly, is not exclusive to anger, as it can also symbolize love (Jonauskaite et al., 2020a). Fugate and Franco (2019) highlight the low specificity and consistency of hue-emotion associations. Part of this inconsistency and lack of specificity may be attributed to cultural variations (Jonauskaite et al., 2020a) but part remains unexplained.

### 1.7 Text-to-image models

Research on color-emotion associations typically relies on words and color patches to elicit emotional responses. While these controlled experiments ensure precision, they limit external validity in real-world settings. On the other hand, studies using specific images are more difficult to control and are often restricted to facial expressions. Text-to-image models offer new possibilities for investigating color-emotion associations.

Text-to-image models are deep neural networks trained on billions of image-description pairs with the goal of learning a joint representation that allows for the generation of images from descriptive text (Ko et al., 2023). Text-to-image models commonly use an architecture known as a diffusion model, which is trained to reconstruct an image, distorted by noise, based on a descriptive prompt (Croitoru et al., 2023). The learning process is nonlinear and approximates a mathematical structure known as a manifold (Chollet, 2021, chapter 2). In the case of text-to-image models, the manifold represents the underlying relationships between language and images, enabling the creation of novel, yet valid and plausible, images within the bounds of the learned data (Rombach et al., 2022). It is important to note that the manifold generalization is confined to the training data, meaning the generated images reflect central trends and patterns in image-text relationships in a non-linear way, and cannot generate images that are out of the image-text distribution learned (Chollet, 2021). Furthermore, the image generation process begins from random seeds of noise, allowing for the random sampling of images.

While traditional experimental methods have established important links between color properties and emotional responses, emerging technologies such as text-to-image models offer new, expansive vistas for exploring color-emotion associations. The present research aims to determine what color-emotion associations are captured in a text-to-image model, with the goal of enhancing our general understanding of color-emotion associations. The study additionally focuses on the role of dominance and the interactions between color dimensions and emotional dimensions in color-emotion associations.

## 2 Materials and methods

Two experiments were conducted. In both, emotion was the independent variable, image subject was the random independent variable, and color features of the produced images were the dependent variables. To operationalize the independent variable, prompts in a text-to-image model were created using the structure *EMOTION WORD + SUBJECT WORD*. For example, for the emotion word *scared* and the subject *tree*, the prompt used was “scared tree.” Each prompt generated a group of four random image replications for each condition. The text-to-image model employed was Openart-SDXL (OpenArt, 2024), a variant of Stable Diffusion XL (Podell et al., 2023), which is a state-of-the-art text-to-image model.

Experiment 1 involved eight emotion words representing high and low levels of valence, arousal, and dominance, in a 2^3^ design. The words were selected from the NRC VAD lexicon (Mohammad, 2018) to represent extreme terms for each desired emotional combination. The NRC VAD lexicon assigned values of valence, arousal and dominance to words, ranging from zero to one, based on human ratings. Its performance is similar or superior to the lexicon developed for Warriner et al. (2013).

Table 1 shows the selected words along with their VAD lexicon values and associated emotional combinations.

**Table 1:** Words selected to represent combinations of high/low valence, arousal and dominance.

Word	Valence	Arousal	Dominance
Victorious	High (0.884)	High (0.886)	High (0.946)
Joking	High (0.776)	High (0.839)	Low (0.407)
Reliable	High (0.912)	Low (0.241)	High (0.875)
Soft	High (0.760)	Low (0.179)	Low (0.232)
Beastly	Low (0.146)	High (0.874)	High (0.843)
Scared	Low (0.146)	High (0.828)	Low (0.185)
Finalized	Low (0.245)	Low (0.210)	High (0.608)
Bored	Low (0.153)	Low (0.167)	Low (0.196)

Values of the emotional dimension, ranging from zero (low) to one (high) are in parentheses.

Since color-emotion associations may vary depending on the subject, according to in-context theory, 21 different subjects were selected. The goal was to include 10 abstract and 10 concrete subjects, along with a representation of emotion without subject. The selection of subjects was inspired by the semantic domains of the Intercontinental Dictionary Series and its entries (IDS) (Key and Comrie, 2021) but was not strictly mapped one-to-one to IDS domains. IDS comprises 22 semantic domains and 1,330 lexical entries that are expected to be cross-cultural and common to human experience. We verified the abstractness/concreteness of the subjects using the lexicon of Brysbaert et al. (2014). Brysbaert et al. (2014) crowdsourced human ratings of concreteness for 40.000 English lemmas in a scale from one (abstract) to five (concrete), generating one of the most widely used resources of concreteness for English. Table 2 lists the 21 selected subjects and their associated IDS domains, where applicable along with concreteness ratings.

**Table 2:** Selected subject words with its correspondent IDS domain and concreteness rating.

Word	Associated IDS domain	Concreteness rating
Water	The physical world	5
Baby	Kinship	5
Man	Kinship	4.79
Woman	Kinship	4.46
Horse	Animals	5
Neck	The Body	5
Apple	Food and Drink	5
House	The House	5
Tree	Agriculture and Vegetation	5
Ball	Motion	5
Tax	Possession	3.89
Space	Spatial Relations	3.54
Quantity	Quantity	2.97
Time	Time	3.07
Thought	Cognition	1.97
Language	Speech and Language	2.35
Politics	Social and Political relations	2.66
War	Warfare and Hunting	3.63
Law	Law	2.57
Religion	Religion and Belief	1.71
Abstract representation	N/A	N/A

IDS, Intercontinental Dictionary Series. Concreteness values ranging from zero (low) to five (high).

Sample sizes were calculated using the R library sjstats 0.19.0 (Lüdecke, 2024). Sample sizes were calculated for a mixed model of only two hierarchical levels, an effect size of 0.3, power of 90%, with 21 subjects. This resulted in four replications per subject per combination of levels, and a total sample size of 672 images generated for Experiment 1 and 504 for Experiment 2.

For color, four dependent variables were selected: Chroma, Lightness (L*), a* (red-green vector), and b* (yellow-blue vector), all derived from the CIELAB standard (CIE, 2007). CIELAB was selected because is a widely used uniform color space where Euclidean distances are meaningful from a perceptual point of view. Therefore, differences in color variables can be calculated through standard statistical analyses and can be interpreted as potential perceptual differences. The values for color variables in each images were obtained using the Python library scikit-image (Van der Walt et al., 2014).

Experiment 2 retained the 21 subjects and the four color variables but changed the emotion descriptors to reflect the well-known Ekman labeling of six basic discrete emotions (Ekman, 1971). Ekman’s labels were adjusted to fit the subject (e.g., “angry” instead of “anger,” “disgusted” instead of “disgust,” “fearful” instead of “fear,” “happy” instead of “happiness,” “sad” instead of “sadness,” and “surprised” instead of “surprise”).

For the analysis of the two experiments, we ran a full mixed model for each dependent color variable, using valence, arousal, and dominance as factors in Experiment 1, and discrete emotions as the factor in Experiment 2. In both experiments, subject was treated as a random intercept. For *post-hoc* tests in Experiment 1, specific contrasts adjusted for false discovery rate (Benjamini and Hochberg, 1995) with α = 0.01 were obtained. In Experiment 2, pairwise Tukey differences with α = 0.01 were calculated.

## 3 Results

### 3.1 Experiment 1

Table 3 summarizes the results of the mixed linear model for the fixed effects of chroma and lightness while Table 4 presents the results for the fixed effects on a* and b*. Table 5 displays the effect of the random intercept for the four color variables.

**Table 3:** Fixed effects of the mixed model of Chroma and lightness explained by valence, arousal and dominance.

Predictor	Chroma				Lightness			
	*β*	SE	*t*(644)	*p*	*β*	SE	*t*(644)	*p*
Valence	**2.74**	**0.79**	**3.47**	**<0.001**	**7.94**	**1.50**	**5.29**	**<0.001**
Arousal	−0.31	0.79	−0.38	0.70	**−14.7**	**1.50**	**−9.78**	**<0.001**
Dominance	−0.92	0.79	−1.16	0.25	−1.22	1.50	−0.82	0.41
Valence*Arousal	1.49	1.12	1.33	0.18	**6.17**	**2.12**	**2.91**	**<0.01**
Valence*dominance	−0.79	1.12	−0.70	0.48	**−9.21**	**2.12**	**−4.34**	**<0.001**
Arousal*dominance	**2.79**	**1.12**	**2.49**	**0.01**	2.64	2.12	1.24	0.21
Valence*arousal*dominance	1.08	1.58	0.68	0.49	−0.19	3.00	−0.06	0.95

Bold formatting denotes statistical significance.

**Table 4:** Fixed effects of the mixed model of a* and b* explained by valence, arousal and dominance.

Predictor	a*				b*			
	*β*	SE	*t*(644)	*p*	*β*	SE	*t*(644)	*p*
Valence	**1.63**	**0.53**	**3.05**	**<0.01**	**1.76**	**0.75**	**2.35**	**<0.05**
Arousal	0.21	0.53	0.40	0.69	**−1.73**	**0.75**	**−2.31**	**<0.05**
Dominance	0.74	0.53	1.40	0.16	−1.40	0.75	−1.86	0.06
Valence*Arousal	0.92	0.75	1.23	0.22	0.67	**1.06**	0.63	0.53
Valence*dominance	**−2.97**	0.75	**−3.94**	**<0.001**	−1.72	**1.06**	−1.62	0.11
Arousal*dominance	**2.02**	**0.75**	**2.68**	**<0.01**	0.28	1.06	0.26	0.79
Valence*arousal*dominance	1.82	1.07	1.71	0.08	1.90	1.49	1.27	0.20

Bold formatting denotes statistical significance.

**Table 5:** Random intercept of the mixed model for Chroma, lightness, a* and b*.

Color variable	Subject random effect		
	Likelihood ratio test	*p*	% of subject variance
Chroma	90.75	<0.001	18.73%
Lightness	161.40	<0.001	28.25%
a*	251.56	<0.001	38.39%
b*	246.07	<0.001	37.82%

**Figure 1:**
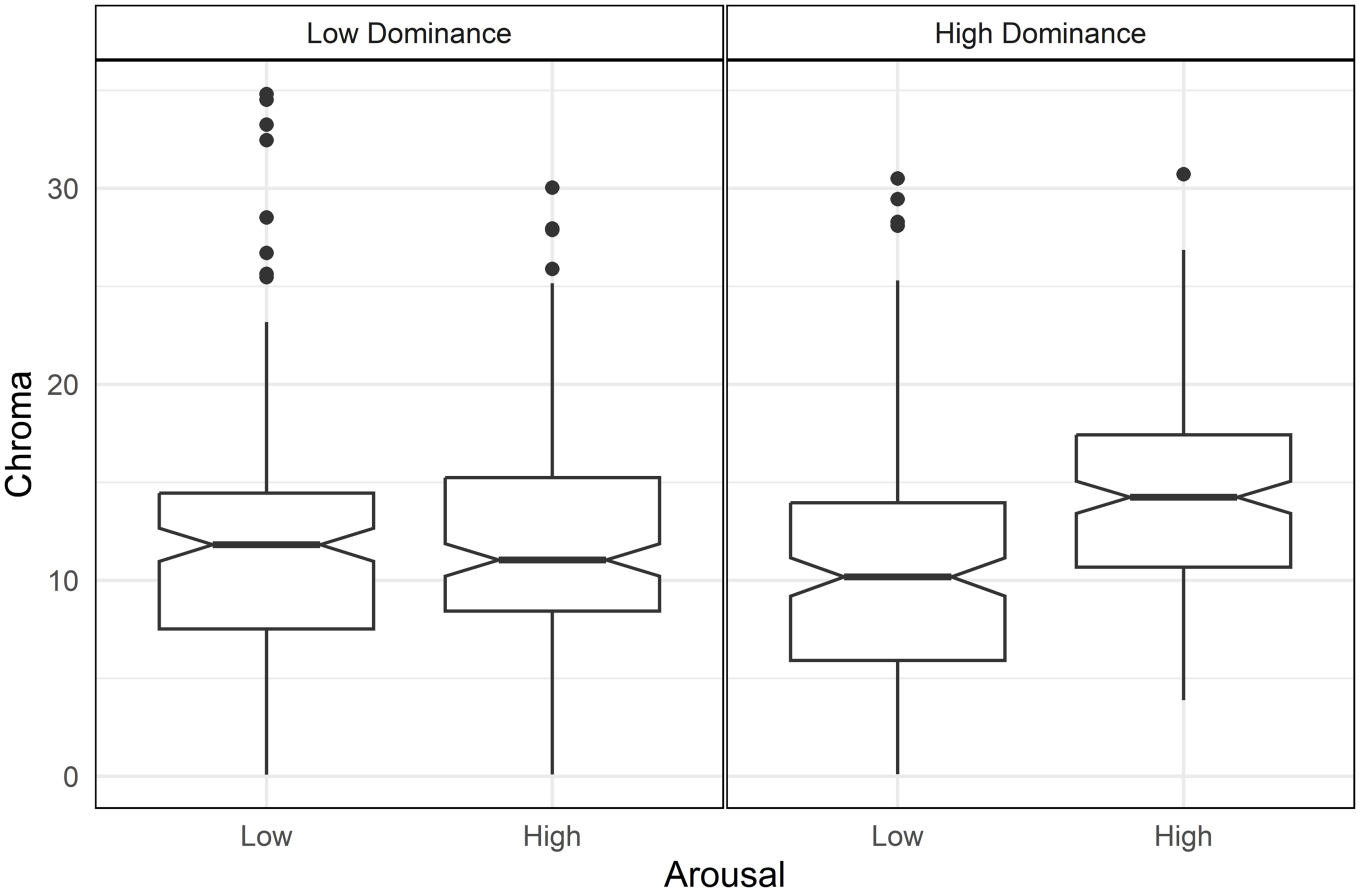
Boxplots of Chroma by arousal and dominance. Only high dominance (right panel) shows a difference in Chroma between arousal levels.

The analysis showed that higher valence increased chroma but with an interaction between arousal and dominance (Table 3). Specifically, arousal increased chroma but only under conditions of high dominance[d = 0.74, *t*(644) = 6.72, *p* < 0.001] (Figure 1).

**Figure 2:**
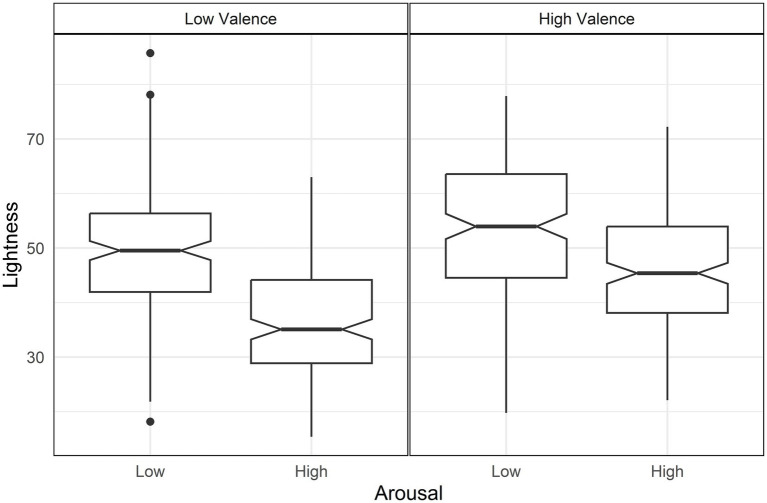
Boxplots of lightness by arousal and valence. Arousal differences in lightness are higher in low valence (left panel).

**Figure 3:**
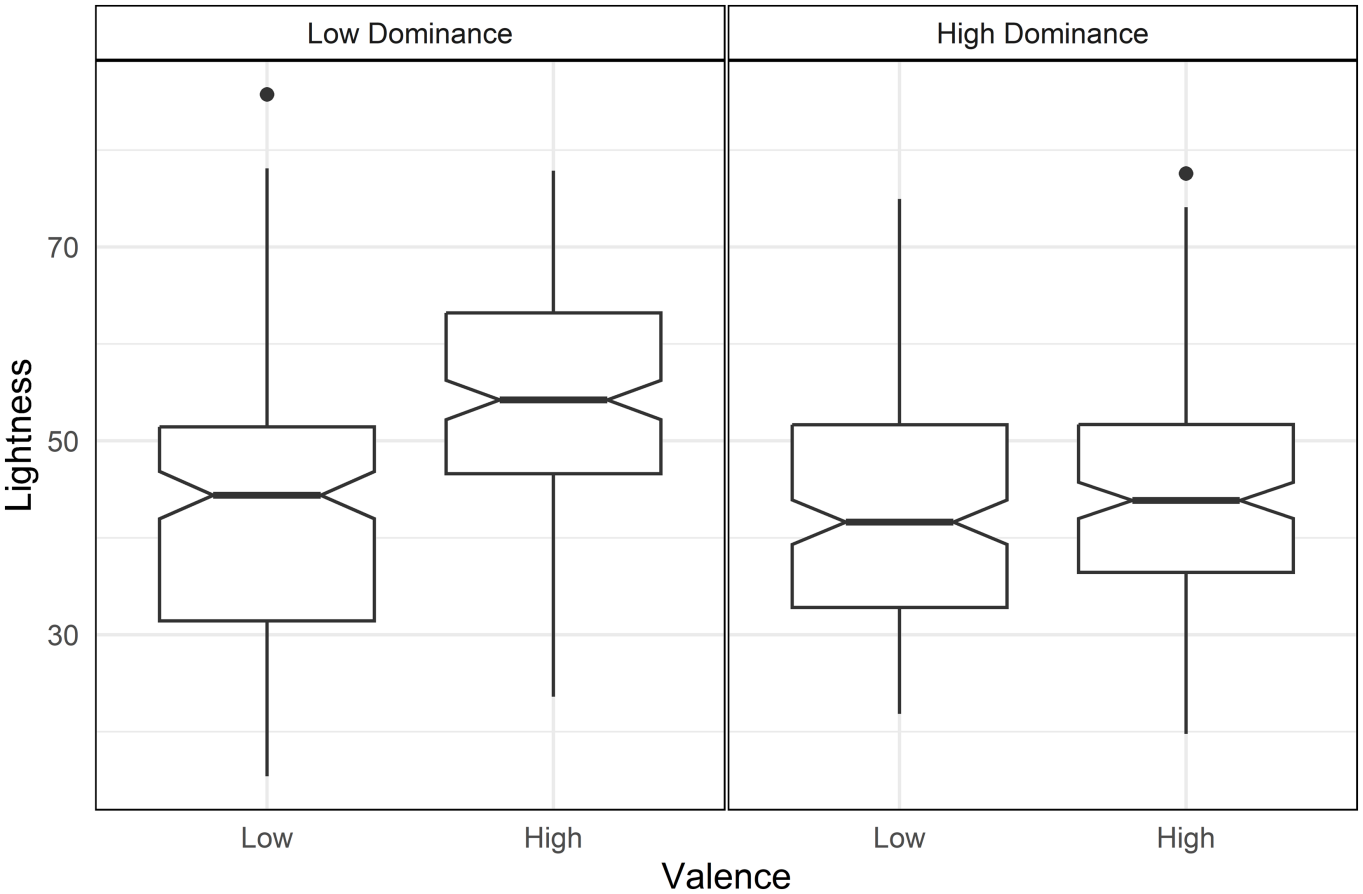
Boxplot of lightness by valence and dominance. High valence and low dominance (left panel) is higher in lightness than the other conditions.

For lightness, valence and arousal had opposite effects: higher valence increased lightness while higher arousal decreased it (Table 3). As a result, the highest lightness is observed in high valence and low arousal, whereas the lowest lightness occurred in conditions of low valence and high arousal (Figure 2). This outcome is further moderated by two interactions: valence with arousal and valence with dominance. The first interaction indicated that the contrast between valence and arousal in lightness was more pronounced at low levels of valence [d = 0.63, *t*(644) = 4.05, *p* < 0.001] (Figure 2). The second interaction showed that lightness increased particularly for high valence when combined with low dominance over all other conditions (Figure 3), with the following differences: Compared with low valence and low dominance, d = 1.13, *t*(644) = 10.39, *p* < 0.001; compared with low valence and high dominance [d = 1.12, *t*(644) = 10.30, *p* < 0.001]; compared with high valence high dominance [d = 0.95, *t*(644) = 9.21, *p* < 0.001].

**Figure 4:**
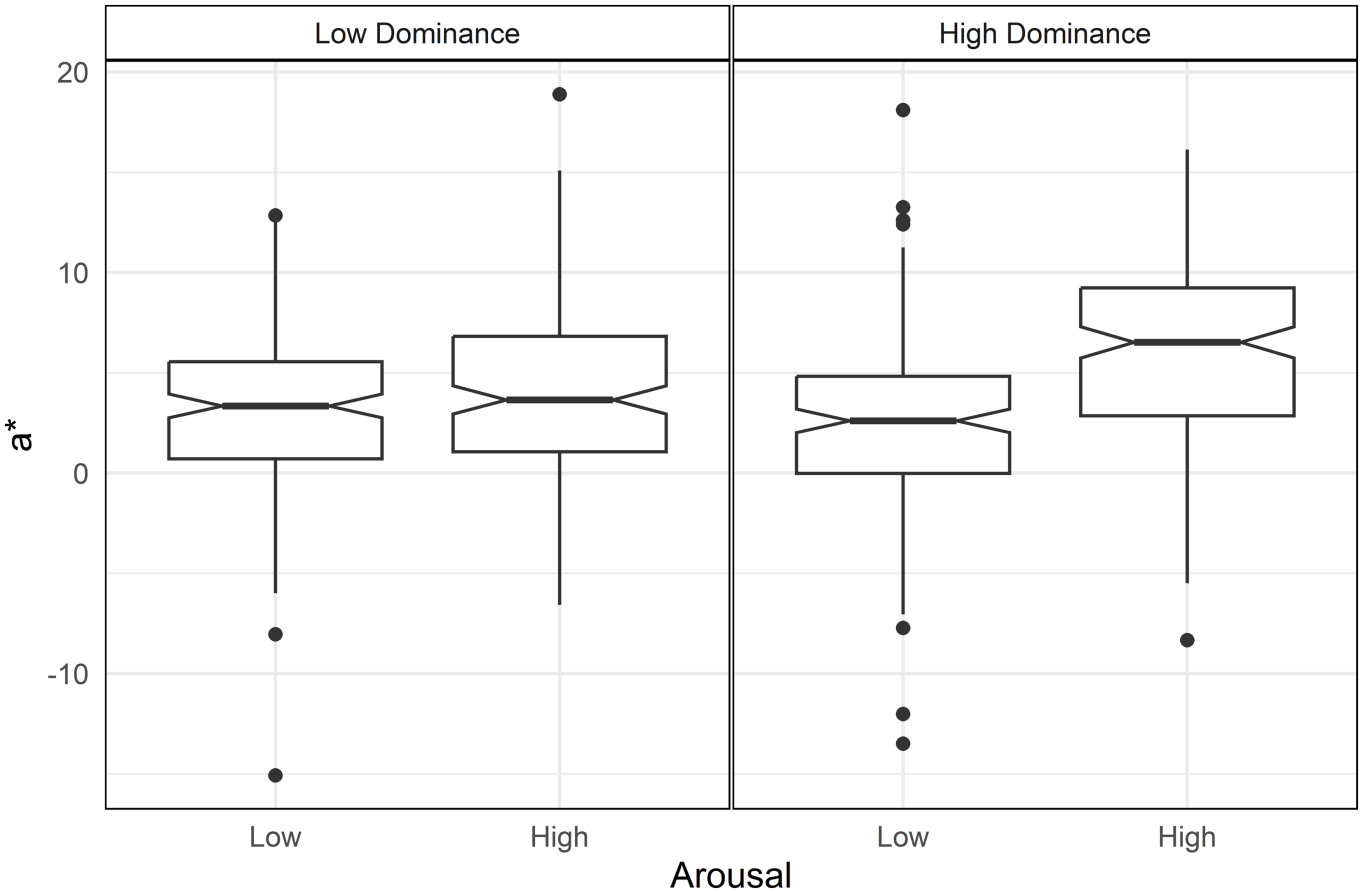
Boxplot of a* (red-blue axe) by arousal and dominance. Only high dominance (right panel) shows a difference in a* between arousal levels.

Higher valence increased the a* value, shifting it toward red (Table 4). This effect was moderated by interactions between arousal and dominance, as well as valence and dominance. The arousal-dominance interaction showed that arousal increased redness only under conditions of high dominance, following a pattern similar to the chroma interaction [d = 1.04, *t* = 9.56, *p* < 0.001] (Figure 4).

**Figure 5:**
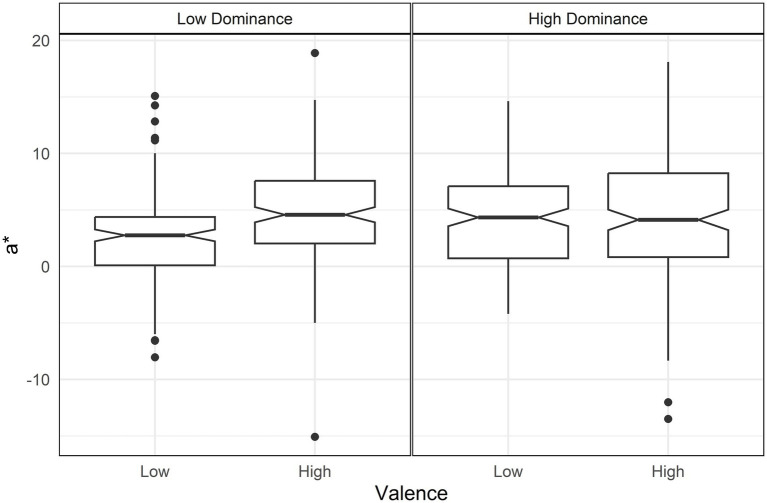
Boxplot of a* (red-blue axe) by valence and dominance. Low valence and low dominance (left panel) is lower in a* than the other conditions.

The valence-dominance interaction indicated that conditions of low valence combined with low dominance resulted in lower levels of red (Figure 5). Against high valence low dominance (d = 0.60, *t* = 5.54, *p* < 0.01); against low valence high dominance [d = 0.51, *t*(644) = 4.64, *p* < 0.01]; against high valence high dominance [d = 0.52, *t*(644) = 4.72, *p* < 0.01].

There were no significant effects in the yellow-blue axis (b* value) (Table 4).

Finally, the effect of subjects was clearly significant (Table 5), indicating the importance of controlling for subjects due to their variability in all color variables: chroma, lightness, a*, and b*. For example, “water” had the lowest average b* value (blue), whereas “horse” had the highest (yellow).

**Figure 6:**
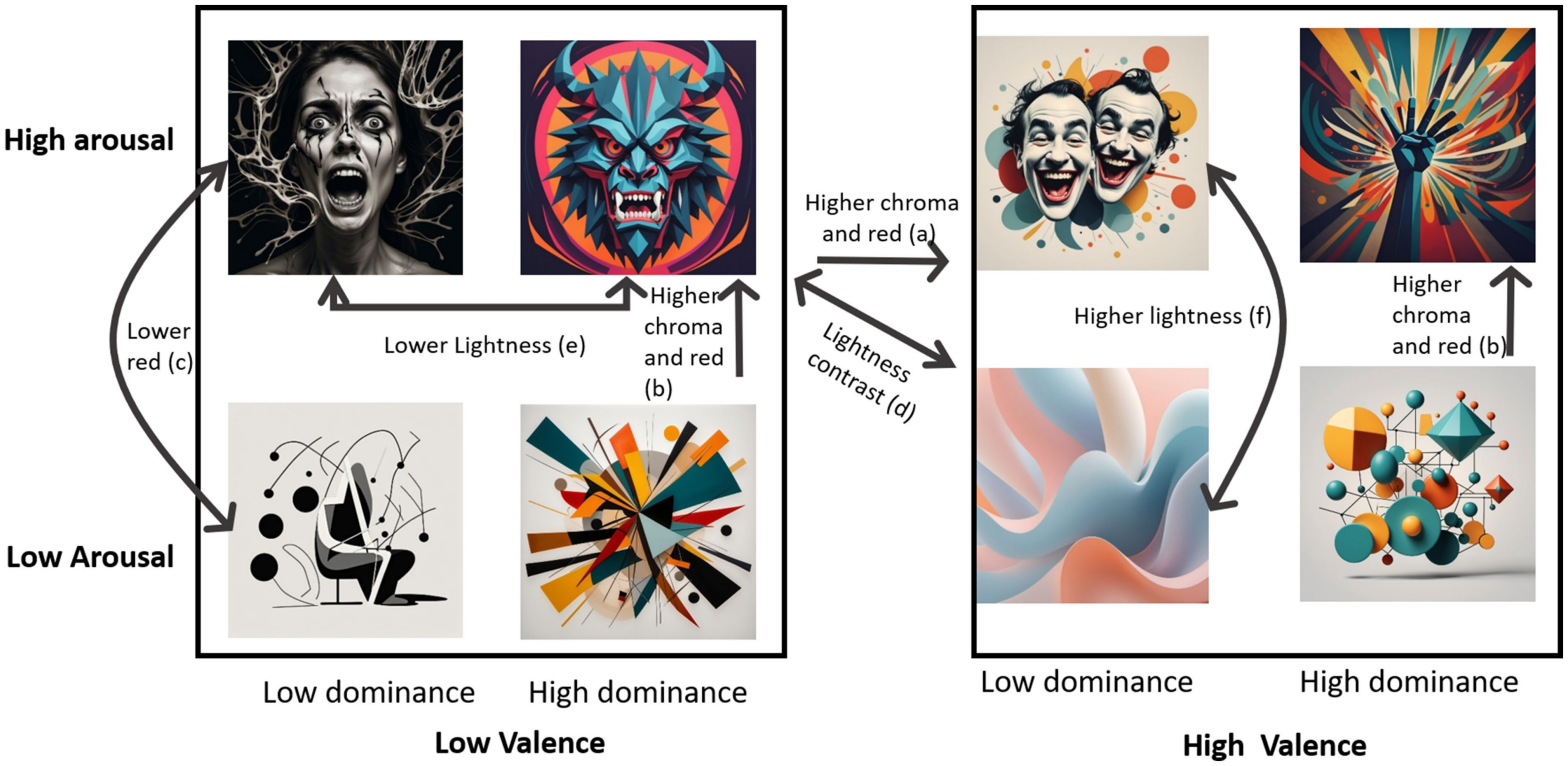
Summary of significant effects of valence, arousal and dominance in Chroma, lightness, a* and b*.

A summary of results is shown in Figure 6. Valence jointly increased red and chroma (a); also, high arousal with high dominance generated an uplift in red and chroma (b). Red is lower too in low valence with low dominance (c). Valence and Arousal generated a lightness contrast (d). The arousal contrast of increased darkness in high arousal is even striking in low valence (e). Finally, lightness is particularly high in high valence with low dominance (f).

### 3.2 Experiment 2

**Figure 7:**
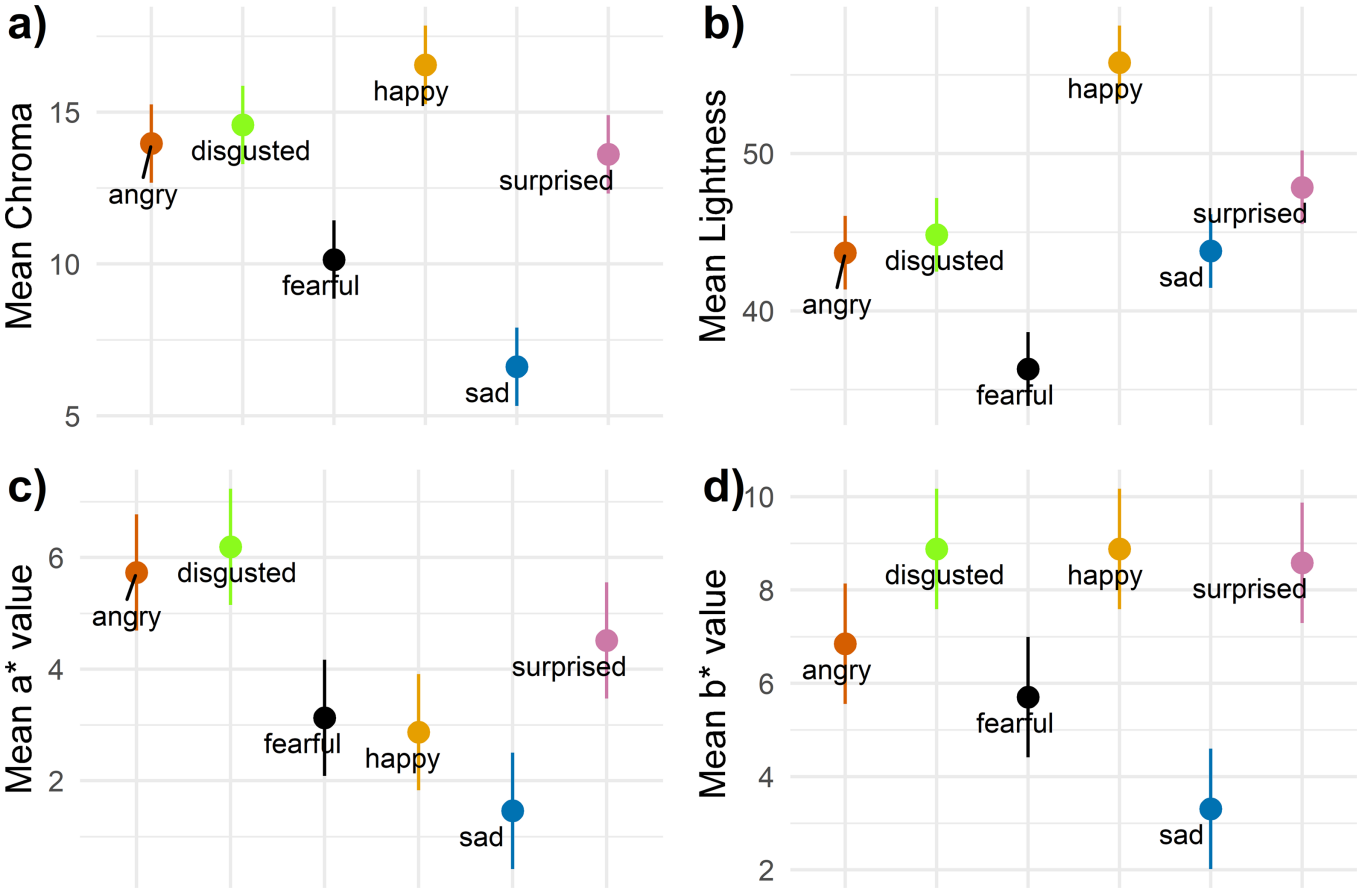
95% confidence intervals for discrete emotions in **(a)** Chroma, **(b)** lightness, **(c)** a* value and **(d)** b* value.

Discrete emotions in the prompt significantly influenced image chroma. *Post-hoc* tests revealed that *sad* had lower chroma than the other five emotions, while *fearful* had higher chroma than *sad* but lower that the remaining four emotions (Figure 7a and Table 6).

**Table 6:** Tukey pairwise comparisons among emotions for Chroma and lightness.

Comparison	Chroma				Lightness			
	d	SE(d)	*t*(498)	*p*	d	SE(d)	*t*(498)	*p*
Angry-disgusted	−0.10	0.154	−0.66	0.99	−0.10	0.154	−0.68	0.57
Angry-fearful	**0.64**	**0.156**	**4.11**	**<0.001**	0.68	**0.156**	**4.38**	**<0.001**
Angry-happy	−0.43	0.155	−2.79	0.0606	**−1.10**	**0.158**	**−7.16**	**<0.001**
Angry-sad	**1.22**	**0.159**	**7.92**	**<0.001**	−0.01	0.154	−0.06	0.95
Angry-surprised	0.06	0.154	0.38	0.99	−0.38	0.155	−2.46	0.02
Disgusted-fearful	**0.73**	**0.156**	**4.78**	**<0.001**	**0.78**	**0.156**	**5.06**	**<0.001**
Disgusted-happy	−0.33	0.155	−2.13	0.27	**−1.00**	**0.158**	**−6.49**	**<0.001**
Disgusted-sad	**1.32**	**0.160**	**8.58**	**<0.001**	0.09	0.154	0.61	0.58
Disgusted-surprised	0.16	0.154	1.04	0.90	−0.27	0.155	−1.79	0.09
Fearful-happy	**−1.06**	**0.158**	**−6.90**	**<0.001**	**−1.78**	**0.164**	**−11.54**	**<0.001**
Fearful-sad	**0.59**	**0.155**	**3.80**	**0.002**	**−0.69**	**0.156**	**−4.45**	**<0.001**
Fearful-surprised	**−0.58**	**0.155**	**−3.73**	**0.003**	**−1.06**	**0.158**	**−6.84**	**<0.001**
Happy-sad	**1.65**	**0.163**	**10.71**	**<0.001**	**1.09**	**0.158**	**7.10**	**<0.001**
Happy-surprised	0.49	0.155	3.17	0.02	**0.73**	**0.156**	**4.70**	**<0.001**
Sad-surprised	**−1.16**	**0.159**	**−7.53**	**<0.001**	−0.37	0.155	−2.40	0.02

SE(d), Standard error of the effect size *d*. Bold formatting denotes statistical significance.

Discrete emotions also affected image lightness. *Post-hoc* tests indicated that *happy* had the highest lightness among the six emotions, while *fearful* had the lowest. *Surprised* was also higher in lightness than *angry* (Figure 7b and Table 6).

For the a* value, discrete emotions showed distinct effects. *Angry* and *disgusted* produced higher levels of red compared to *fearful*, *happy*, and *sad*, though not compared to *surprised*. Additionally, *surprised* had higher red levels than *sad* (Figure 7c and Table 7).

**Table 7:** Tukey pairwise comparisons among emotions for a* and b*.

Comparison	a*				b*			
	d	SE(d)	*t*(498)	*p*	d	SE(d)	*t*(498)	*p*
Angry-disgusted	−0.09	0.154	−0.61	0.58	−0.34	0.155	−2.19	0.04
Angry-fearful	**0.54**	**0.155**	**3.48**	**<0.01**	0.19	0.154	1.23	0.27
Angry-happy	**0.59**	**0.155**	**3.82**	**<0.001**	−0.34	0.155	−2.19	0.04
Angry-sad	**0.88**	**0.157**	**5.70**	**<0.0001**	**0.59**	**0.155**	**3.81**	**<0.001**
Angry-surprised	0.25	0.155	1.63	0.12	−0.29	0.155	−1.87	0.09
Disgusted-fearful	**0.63**	**0.156**	**4.09**	**<0.001**	**0.53**	**0.155**	**3.42**	**<0.01**
Disgusted-happy	**0.68**	**0.156**	**4.43**	**<0.001**	0.00	0.154	0.00	0.99
Disgusted-sad	**0.97**	**0.157**	**6.31**	**<0.001**	**0.93**	**0.157**	**6.00**	**<0.0001**
Disgusted-surprised	0.35	0.155	2.24	0.04	0.05	0.154	0.32	0.80
Fearful-happy	0.05	0.154	0.34	0.73	**−0.53**	**0.155**	**−3.42**	**<0.01**
Fearful-sad	0.34	0.155	2.22	0.04	0.40	0.155	2.57	0.02
Fearful-surprised	−0.29	0.155	−1.85	0.08	**−0.48**	**0.155**	**−3.10**	**<0.01**
Happy-sad	0.29	0.155	1.88	0.08	**0.93**	**0.157**	**6.00**	**<0.001**
Happy-surprised	−0.34	0.155	−2.19	0.04	0.05	0.154	0.32	0.80
Sad-surprised	**−0.63**	**0.156**	**−4.07**	**<0.001**	**−0.88**	**0.157**	**−5.68**	**<0.001**

SE(d), Standard error of the effect size *d*. Bold formatting denotes statistical significance.

Lastly, discrete emotions influenced the b* value, though the effects were smaller. *Disgusted* and *happy* showed higher levels of yellow than *fearful* and *sad*, while *surprised* and *angry* only showed higher yellow levels than *sad* (Figure 7d and Table 7).

Overall, *sad* and *fearful* had low values across all four color variables, with *fearful* showing the lowest lightness and *sad* having the lowest chroma, a*, and b* values. In contrast, *happy* had the highest levels of chroma and lightness. It also exhibited high yellowness but lower red levels. *Surprised* also displayed high lightness and yellowness. High red levels and low lightness characterized *angry*, while *disgusted* showed high chroma, red, and yellow values.

### 3.3 Robustness

In order to assess robustness of the results to extreme values, analyses were replicated using robust linear mixed models and robust *post-hoc* tests. All the main results of Experiment 1 held and there were some minor differences in the red level of anger and surprise in experiment 2. Such change did not affect the presented results. Robust analyses are included as supplementary material in the link at the data availability statement.

## 4 Discussion

Emotional images were generated using a text-to-image model to detect potential color patterns associated with the emotions depicted. The results show significant associations across subjects, with chroma, lightness, and red strongly associated with emotion dimensions and specific emotions, along with several interactions between the emotional dimensions.

The findings highlight three key elements for discussion: (1) the joint role of red and chroma, (2) the role of lightness in depicting emotions and (3) specific color element combinations for concrete emotions.

### 4.1 The role of red and Chroma

The evidence supports a joint role of red and chroma in the depiction of emotion, with three main effects.

The results suggest that the combined presence of both chroma and redness is necessary to effectively convey emotions characterized by high arousal and high dominance, regardless of valence. Previous studies have linked both arousal and dominance to red and chroma/saturation (see the reviews of Elliot, 2015, 2019). However, red has also been found to lack specificity, and can be associated to both, positive and negative emotions (Fugate and Franco, 2019; Jonauskaite et al., 2020a). Findings of the research contribute to this body of work by identifying two distinct valence-related effects involving red and chroma. First, increased red and chroma tend to depict positive emotions, and the effect is intensified in dominant emotions with high arousal like triumph and pride. This effect is termed the *joy effect*. Second, high chroma and red also play a key role in negative, high-arousal, high-dominance emotions, such as anger, named as a *rage effect*.

This finding may help explain previous discrepancies in the literature, where saturation without red has been associated with low arousal (Weijs et al., 2023), and where red without high saturation may not strongly convey dominance (Stephen et al., 2012). It also sheds light on conflicting findings regarding saturation as an indicator of arousal only in low lightness conditions (Weijs et al., 2023; Oh and Park, 2022) or only in high lightness contexts (Wilms and Oberfeld, 2018). The results show that saturation, when combined with chroma, signals arousal in both high and low lightness conditions, as the associated emotional states consistently exhibit high arousal and dominance, independent of valence.

Conversely, low-valence, low-dominance emotions such as boredom, sadness, and fear, are typically associated with low chroma and low red, or are entirely achromatic (*negative powerless effect*). These findings align with prior work by Kaya and Epps (2004). Supporting evidence from Bekhtereva and Müller (2017) suggests that color scenes enable higher-level emotional processing compared to grayscale ones, implying that achromatic representations may correspond to low emotional intensity across dimensions. Furthermore, studies in faces have shown that pale faces might imply fear (Montoya et al., 2005) and submission (Setchell and Jean Wickings, 2005), reinforcing the link between achromaticity and emotional powerlessness.

### 4.2 The role of lightness

The evidence also points to a clear role of lightness in emotion representation. Although the role of lightness as emotionally positive and darkness as emotionally negative are well stablished (Lakens et al., 2013; Okubo and Ishikawa, 2011; Jonauskaite and Mohr, 2025), the present research refines such results with three distinct effects. First, a *stress/relief divergence*, where high lightness is associated with relief emotions (high valence, low arousal), while low lightness corresponds with stress-related emotions (low valence, high arousal). This finding extends the results obtained previously in virtual reality (Weijs et al., 2023) and environmental color research (AL-Ayash et al., 2016; Oh and Park, 2022). Second, *a threat effect*, where in low-valence conditions, lower lightness in high-arousal emotions is more pronounced than in low-arousal emotions. Thus, emotions such as fear and anger are characterized by greater darkness than their low-arousal counterparts such as boredom (Jonauskaite and Mohr, 2025), which are more gray (Demir, 2020). Finally, high lightness is associated with emotions of high valence and low dominance, such as calm, awe, and gratitude, a phenomena scarcely reported in the literature (Valdez and Mehrabian, 1994; Jonauskaite et al., 2020b). These emotions involve low control over a situation but retain a positive valence, a phenomenon labeled the *serenity effect*.

Collectively, these effects emphasize the importance of dominance as a moderating variable in color-emotion associations, a dimension that has also been shown to play a significant role in semantic cross modal correspondences (Alvarado et al., 2023), even across cultures (Alvarado, 2024).

### 4.3 Specific emotion combinations

These six identified effects can be observed in the emotional profiles of the specific categories analyzed in Experiment 2.

Anger incorporates the *rage* effect, *stress/relief divergence*, and the *threat* effect, resulting in a combination of chromatic red and darkness. Fear and Sadness combine the *threat* effect and the *negative powerless effect*, producing a mix of darkness and low chromaticity. Thus, red helps to separate anger from fear (Young et al., 2013) and anger can be both, red and black (Jonauskaite et al., 2020a). The *stress/relief divergence*, combined with the *rage* and *threat effects*, help explain the increased heart rate found in previous studies when low lightness and high chroma are present (Oh and Park, 2022; Weijs et al., 2023). This combination of color elements evokes emotions like threat, rage, and stress, which elicit a defensive, urgent response.

Surprise sits in an intermediate position for all color variables, as it can have both positive and negative valence, mixing the *joy* effect and the *negative powerless* effect. Finally, happiness stands out with the highest levels of lightness and chroma, consistent with the *joy* effect, though its red levels are lower than expected.

However, disgust did not exhibit all the expected effects. Based on its low valence, high arousal and low dominance, one might expect disgust to behave similarly to fear—achromatic and dark. While it shares the low lightness typical of the *threat* effect, its chromaticity is high in both red and yellow, not exhibiting the *negative powerless* effect. This suggests that undiscovered specific effects might be influencing the color-emotion associations for disgust.

Overall, results highlight the importance of combinations of color dimensions in the generation of images for specific emotions, regardless of the depicted subject.

Interestingly, hue effects, beyond red, were not strongly observed. For example, no consistent association between sadness and blue emerged, echoing previous findings (Schloss et al., 2020) and no significant trends were found along the yellow-green axis (b*). This suggests that hue may play a more central role in object or source identification (Goubet et al., 2018; Spence, 2020), limiting its capacity to express emotion—except in the case of red. In contrast, lightness and saturation appear to be the primary carriers of emotional meaning across contexts.

### 4.4 Implications for emotion artificial intelligence (emotion AI)

With the growing integration of artificial intelligence in creative and interactive systems based in artificial intelligence, the ability to understand and convey emotions has become a key feature of Emotion AI. Findings of the current research offer concrete insights into how specific combinations of color properties—particularly chroma, lightness, and red hue—map onto emotional dimensions like arousal, dominance, and valence. These insights can be directly applied to enhance both the interpretation and generation of emotional content by AI systems.

One important application lies in prompting and generative design. The results can inform more precise prompting strategies in artificial intelligence generative models, whether the goal is to evoke specific emotional effects through visual features or to generate emotionally appropriate visuals from affective prompts. Digital creators and artists working with AI tools could benefit from the typology of effects (e.g., joy effect, rage effect, serenity effect) as a practical palette for affective visual generation and evaluation. For instance, a prompt intended to soothe could emphasize high lightness and desaturated tones.

Additionally, the findings can support more informed labeling in AI training pipelines. Many visual emotion recognition systems depend on labeled image-emotion pairs, but current labels often overlook the affective contribution of color properties. Including structured labels for saturation, lightness, and some hues, grounded in the presented results, can facilitate more consistent labeling practices and help models better learn the affective structure of visual scenes. This may be especially relevant for training multimodal systems that must infer emotional meaning from both object content and ambient visual tone understanding, for example, that a combination of high chroma and red yields emotionally intense, dominant visuals—ideal for expressions of anger or triumph—while high lightness with low chroma better captures calm or gratitude.

Finally, interactive AI systems such as avatars, video games, or emotionally adaptive interfaces can deploy the findings to improve emotional communication and immersion. By dynamically adjusting environmental or character-related color properties according to the emotional content of a scenario—or the user’s inferred emotional state—AI systems can deliver more emotionally resonant experiences. For example, a digital character designed to express stress could combine low lightness with high chroma red, while a moment of triumph might be characterized by high lightness with high chroma red.

In summary, the present work provides a structured framework for linking color properties to emotional content, with direct implications for how AI systems generate, label, and interact through emotionally expressive visuals. These contributions lay the foundation for more emotionally literate and context-sensitive artificial intelligence.

### 4.5 Overall results, limitations, and future research

Text-to-image models show specific red, chroma and lightness associations with emotions regardless of the subject of the image. Since text-to-image models rely on a compressed neural representation of a vast collection of images, it can be argued that the outcomes reveal clear general patterns in the associations between emotions and color variables.

Beyond simple and direct associations between specific emotions and certain colors, the interactions between emotional dimensions lead to specific effects in color variables, enabling a greater variety and complexity in the associations between emotion and color. Red and chroma effects of joy, rage and negative powerless, jointly with lightness effects of serenity, threat, and stress/relief divergence allow for a range of combinations for representing concrete emotions in a general manner.

Overall, this research contributes to a deeper understanding of how color variables can be used to convey emotions in visual representations, demonstrating the potential of text-to-image models as valuable tools in studying color-emotion relationships.

Future research might extend this work to different text-to-image models to detect similarities of differences between them, and might look for color-emotion associations on specific subjects such as architecture or food. Plenty of possibilities are open in the field of color-emotion associations with the use of text-to-image models.

Text-to-image models have some limitations. Their results are likely biased, further compounded by the difficulty in identifying these biases due to the large volume of images used for training and the inherent opacity of neural network models. Additionally, the generated images reflect the most general trends, often excluding less represented patterns or groups. Nevertheless, it can be expected that they capture at least the most prominent trends found in the images available on the Internet. Future studies might tackle cultural differences and biases in text-to-image models.

In a significant number of images, the text-to-image model included faces to help convey emotions, which may influence the results. Nevertheless, the expressive support provided by the faces did not prevent the emergence of strong general associations between emotions and colors.

## Data Availability

The datasets presented in this study can be found in online repositories. The names of the repository/repositories and accession number(s) can be found at: https://osf.io/hvtqm/?view_only=2df52c699b1b4b4c9ac12bd05dfa0af5.
